# Responses of aerial insectivorous bats to local and landscape-level features of coffee agroforestry systems in Western Ghats, India

**DOI:** 10.1371/journal.pone.0201648

**Published:** 2018-08-16

**Authors:** Shasank Ongole, Mahesh Sankaran, Krithi K. Karanth

**Affiliations:** 1 Post-graduate Program in Wildlife Biology & Conservation, Wildlife Conservation Society – India Program & National Centre for Biological Sciences, Tata Institute of Fundamental Research, Bangalore, Karnataka, India; 2 School of Biology, University of Leeds, Leeds, United Kingdom; 3 National Centre for Biological Sciences, Tata Institute of Fundamental Research, Bangalore, Karnataka, India; 4 Wildlife Conservation Society, New York, New York, United States of America; 5 Centre for Wildlife Studies, Bangalore, Karnataka, India; 6 Environmental Science and Policy, Nicholas School of the Environment, Duke University, Durham, North Carolina, United States of America; Kerala Forest Research Institute, INDIA

## Abstract

Shade coffee has shown great promise in providing crucial habitats for biodiversity outside formal protected areas. Insectivorous bats have been understudied in coffee, although they may provide pest control services. We investigated the influence of local and landscape-level features of coffee farms on aerial insectivorous bats in Chikmagalur district in the Western Ghats biodiversity hotspot, India. Bats were monitored in 20 farm sites using ultrasound detectors, and the response of bat species richness and activity to changes in tree density, proportion of built-up area in the neighborhood, and distance of farm from forest areas quantified. We examined if models built to explain the species richness and activity could also predict them in nine additional sites. We detected nine phonic types/species in the study area. The quantified predictors had no effect on assemblage-level species richness and activity of bats. Responses of edge-space and cluttered-space forager guilds mirrored those of the overall assemblage, but some species vulnerable to forest conversion like *Rhinolophus beddomei* were detected rarely. Best models explained up to 20% and 15% variation in assemblage-level species richness and activity respectively, and were poor predictors of both response variables. We conclude that coffee farms in our study area offer an important commuting space for insectivorous bats across a gradient of shade management. Further research should include species-specific responses to management decisions for at-risk species and quantification of ecosystem services like natural pest control to inform biodiversity conservation initiatives in the Western Ghats coffee landscapes.

## Introduction

Agricultural landscapes, which occupy almost 40% of the planet’s ice-free land area [1] are much more geographically extensive than protected areas. Consequently, they hold immense potential for biodiversity conservation [2–6]. Agroforestry systems—the intentional management of shade trees with agricultural crops—have been shown to be particularly important in this regard due to their structural similarity to forests and significant vegetation diversity [5]. Indeed, studies have shown that in some cases, species richness and composition of many taxa in agroforestry systems are comparable to neighboring forests [7]. Understanding the characteristics of agroforestry systems that allow species to use them, including specific management practices within farms and the landscape context surrounding the farm can therefore inform management strategies that aim to conserve biodiversity outside of protected areas [8]. This understanding can also enhance the provisioning of ecosystem services like biological pest control and pollination [9,10] in these landscapes.

Among agroforestry systems, coffee is the most widespread (grown in over 50 tropical countries) and economically important crop, with a retail value of ~$US90 billion per annum [11,12]. Several studies show that shade-grown coffee holds significant ecological value for various taxa including birds, insects and mammals [3,13–18]. Local-level features of coffee farms, such as type and density of shade trees, and canopy cover have been shown to influence biodiversity in them [8,19,20]. In addition, landscape characteristics surrounding the farms (such as presence of natural forests) have also been shown to be important for biodiversity in the farms [21,22].

The response of insectivorous bats to the local and landscape characteristics in coffee farms remains unclear, even though they hold immense potential for limiting pests in them [10,23]. Research on the impact of land conversion to agriculture or agricultural intensification on bats has largely emerged from the Neotropics [22,24,25] with few Paleotropical studies. [17,26–28]. However, in the last few decades, coffee production has increased in many regions of the Paleotropics with an associated decline in shade tree diversity [29]. At present, the impacts of such changes on Paleotropical bat communities remains poorly understood.

Here, we examine the relative roles of local and landscape-level features in influencing Paleotropical aerial insectivorous bats in coffee agroforests in India’s Western Ghats biodiversity hotspot. Specifically, we asked how local features, such as shade tree density, and landscape features of coffee farms, such as distance to natural forest and proportion of built-up area (buildings) surrounding the farms influence the overall species richness and activity of bats (assemblage-level). We also quantified the responses of different foraging guilds of insectivorous bats—cluttered-space foragers, edge space foragers and open space foragers [30] in these farms (ensemble-level). These ensembles are likely to show different responses because (i) their echolocation call design results in varying ability to forage in cluttered vegetation (ii) their wing morphologies, which influences their maneuverability while capturing insect prey, also result in differing levels of flight efficiencies when commuting over the landscape matrix [31]. In addition, we also examined how species composition of the assemblage was related to the local and landscape-level features. We expected cluttered space forager richness and activity levels to decrease with increasing distance from forest due to their low flight efficiencies and show no response to increasing vegetation clutter at the local level. The response to distance from forest may be weak if the coffee matrix offers low contrast to the forest. We expected species richness and activity levels of edge and open space foragers to increase or show no response to increasing distance from forest. We also expected increasing vegetation clutter at the local level to negatively influence the edge and open space foragers.

## Methods

### Study area and sampled farms

**Fig 1:**
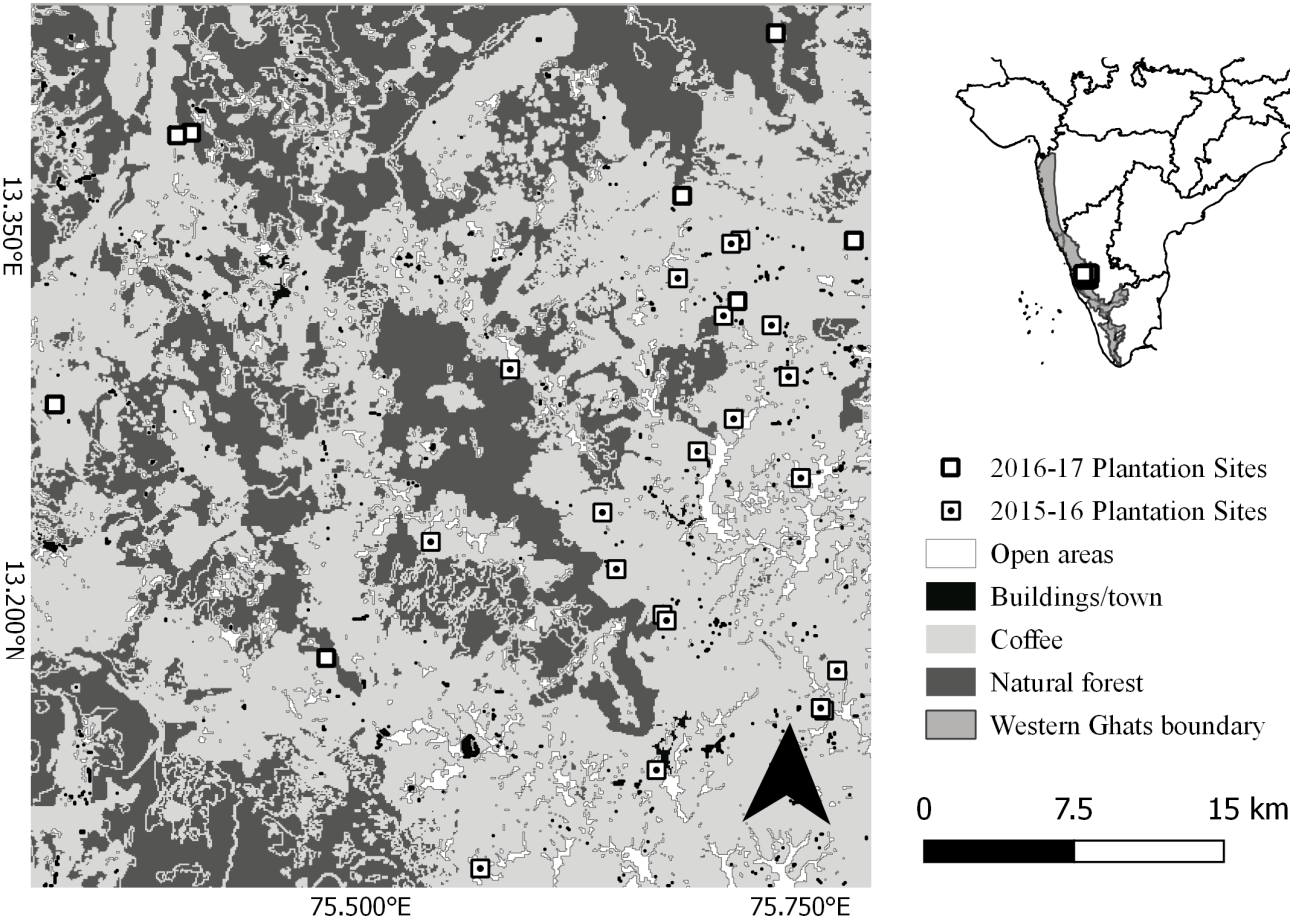
Map of study region. Locations of sampled coffee farms, surrounding forests, built-up and open areas in Chikmagalur district of Western Ghats, India.

We surveyed bats in Chikmagalur district (13°18–13°21´ N and 75°31´-75°48´ E), a significant coffee growing region in the state of Karnataka, India (Fig 1). This region is located within the Western Ghats and Sri Lanka Biodiversity Hotspot [32]. Besides shade coffee cultivation, the study area is comprised of Bhadra Wildlife Sanctuary, a 493 km^2^ tropical moist deciduous forest [33], several reserved and revenue forests [19], agriculture fields and human habitations. Grown at altitudes between 800m and 1500m, coffee cultivation (Arabica variety, *Coffea Arabica*, Robusta variety, *C*.*canephora*) covers an area of over 870 km^2^ in the district and account for ~25% of all coffee produced in India [34]. More than 90% of these comprise small farms with areas <0.1 km^2^. Canopy is largely closed overall with canopy density ranging between 50 and 99 percent [8]. Shade management in farms can lead to changes in tree density and foliage cover. The shade layer can also vary from being dominated by native tree species (e.g. *Ficus glomerata*, *Artocarpus heterophyllus* and *Erythrina indica*) to one dominated by an introduced species from Australia called silver oak (*Grevillea robusta*). At the landscape level, large expanses of monoculture shade are not found as coffee grown under exotic shade is interspersed with that grown under native shade. Even at the local level, exotic shade tree stands are in close proximity to native species. In a pilot study, the percentage of native shade tree species in the study area farms ranged from 24% to 97% (n = 20, mean 50%, sd 21%). Following a scheme based on estimates of shade tree species richness, tree density and shade cover used to classify coffee farms in the Neotropics along an intensification gradient [35], most farms in this region can be classified as low-management coffee.

### Bat surveys

We surveyed bats in 20 coffee farm sites (mean size 5.1 ha, size range from 3.6 to 6.5 ha) in the dry season between November 2015 and April 2016, representing a gradient of shade tree density and located at varying distances from natural forest patches (Fig 1). No wildlife permits were required to monitor bats on these privately owned farms. We obtained verbal permissions from all land owners/managers to sample on their lands. Since we used completely non-invasive acoustic methods to monitor free-flying bats, we did not require approval from an animal ethics committee. The elevations of the sampled sites ranged from 748m to 1267m, with only four sites falling below 1000m. At each site, we acoustically sampled for bats using two Pettersson D240X time expansion bat detectors recording on to Edirol R-09 digital recorders. To account for horizontal spatial variability in bat activity, we sampled at multiple points separated by 100m [36] along narrow walking trails between coffee bushes with three visits to each point across the study duration. However, we recognize that estimates of activity can vary depending on the spatial positioning of detectors even at close distances [37]. Each point in a site was sampled for 15 min in one night between sunset and 22:00 as activity reduced considerably after that. The two detectors were used simultaneously at different points and rotated to the next set of points until all points within the site were exhausted. While sampling for the entire night is a requirement to estimate activity accurately [38], we could not do so due to personnel limitation and note that as a limitation of our study. Detectors were pointed into canopy gaps to maximize detections. Each point in each site was re-surveyed between November 2016 and April 2017, to assess temporal turnover at the assemblage-level. We also sampled an additional 9 sites in 2016–17 (ranging in size from 4 ha to 40 ha) each of which was visited five times during the study period to evaluate models fit with earlier data.

### Characterization of local level vegetation

At each site, we estimated shade tree density using the variable-area transect (VAT) method [39](S1 Fig). We first established multiple 40m baseline transects at each site. Each transect was then divided into four 10m long sub-transects. On either side of these sub-transects, we searched for five shade trees (> = 30 cm GBH) in a rectangular cell of length 10m and width up to 20m. If five trees were found within a distance of 20m, the width was calculated as the distance to the fifth farthest tree. If there were fewer than five trees within 20m, the width was taken to be 20m. Tree density estimates were obtained by dividing the total number of trees counted by the total area of the cells, averaged across all transects. We also estimated a measure of vertical foliage cover by counting the number of height classes from among 2 height classes (between the coffee layer and 10m, 10-20m) that contained leafy vegetation within an imaginary cylinder of 0.5 m radius above the observer, at 20 random points on either side of a transect [19].

### Characterization of landscape level features

Distance of coffee sites from natural forest was estimated using the ISRO Bhuvan Land Use/Land Cover classification layer (1:50000, 2011–2012) of Chikmagalur district (http://bhuvan5.nrsc.gov.in/bhuvan/wms). As the LULC layer was from an earlier time period, we first visually compared the Bhuvan layer with current satellite imagery from Google maps (2017) to determine if forest cover had significantly changed in the last few years. We restricted our analysis to forest patches larger than 1.8 km^2^ as they matched up between the two time periods.

Proportion of builtup area was measured using circular buffers at four radii (100m, 200m, 500m and 1000m) from the centre of each site, to account for the possibility that different bats respond to the landscape at different scales. Radii larger than 1000m were not investigated to avoid overlap between buffers of adjacent sites [40–42]. Overlapping buffers leads to statistical non-independence of observations as the same predictor values are used multiple times in the data set [41]). We used QGIS 2.18.10 [43] to estimate distance to forest areas and Microsoft Bing maps to digitize built-up areas.

### Acoustic data analysis

We used BatSound Pro 3.32 (www.batsound.com) to create spectrograms of the calls (setting for spectrogram: sampling rate 44.1 kHz for time-expanded calls, FFT size = Automatic, FFT Overlap = -1, FFT window = Hanning). Using the power spectrum option, only those with intensity greater than or equal to 20dB above the background noise were used for extracting call structure information (setting for power spectrum: FFT size = Automatic, FFT window = Hanning, Min level = -120 dB). For each call, we extracted frequency of maximum energy (FMAXE), end frequency (EF), start frequency (SF) and call duration (duration). Published echolocation call libraries from the Western Ghats region [44,45] were used to identify bats. Where identification was not possible, we assigned species to phonic types based on similarity of call structure and FMAXE. We classified the detected species/phonic types into guilds based on Denzinger and Schnitzler [46] (2013) by visual inspection of echolocation calls. We labeled species with the following characteristics as cluttered space foragers (i) CF-FM (Constant Frequency-Frequency Modulated) echolocation calls (ii) Broadband calls with low intensity typical of two species expected from the region, *Megaderma spasma* and *M*.*lyra* [47]. We classified species with calls containing a steep, downward frequency modulated component (FM) followed by a shallow, narrowband component (QCF) as edge space foragers. We did not expect to detect edge space trawling foragers as there were no streams and water bodies in the sampling sites. Long, shallow and narrowband echolocation calls were classified as open space foragers.

### Statistical analyses

#### Bat species richness estimation

We estimated species richness at each site using the software SPECRICH2 (https://www.mbr-pwrc.usgs.gov/software/specrich2.shtml). The software accounts for heterogeneity of detection probabilities among species [48] using the jack knife estimator first developed for capture recapture analysis of closed populations [49].

#### Bat activity estimation

We indexed bat abundance based on bat activity as the proportion of the total number of minutes sampled in an estate that yielded any bat call [50]. In order to determine whether detectability of the bat assemblage was similar across sites, we compared average detectability across sites (S1 Dataset). This is a key requirement for comparing activity levels across sites.

#### Model construction

At each scale, we used multiple linear regressions to model the response of assemblage-level bat species richness and activity from 2015–16. We did not expect elevation to have an effect on the bats as the maximum difference in elevation between sites was only 250m. We made pair-wise comparisons between the predictor variables, and only those not highly correlated (r<0.7) were used for analysis. The final set of predictor variables contained tree density, distance from natural forest and proportion of built-up area. To assess whether there is spatial autocorrelation in estimated species richness and activity of bats, we plotted Moran’s I correlograms. For each spatial scale, the candidate model set consisted of all possible combinations of predictors included in a particular model and a model also containing an interaction term between proportion of built-up area and distance from forest for a total of eight models. We included this interaction term as we hypothesized that artificial roosting sites may become increasingly important in agricultural landscapes as distance from natural forests increase. In each model, we standardized each input variable by centring it and then standardizing the result by 2*standard deviations [51]. Centring improves the interpretability of input variables even when interaction terms are present, while standardization makes comparison of effect sizes of multiple input variables possible by placing them on a common scale [52]. Exploratory analysis indicated that the proportion of built-up area for one particular site was an extreme value (0.10) due to its proximity to a large town (median value without the outlier 0.0062). We excluded this observation from our analyses, because this is not representative of a typical coffee farm in the study area. At the ensemble-level, we did not conduct multiple regression analyses for species richness as the data were insufficient. To assess if activity differed at the ensemble-level, we plotted guild-wise activity levels with the predictor variables.

For each response variable at each scale, we followed a model selection approach based on small sample-corrected Akaike’s Information Criterion (AIC_c_) to select the best model (ΔAIC_c_<2) [53]. When there were multiple best models, we estimated model averaged regression coefficients of predictors using the zero method, i.e. if a given predictor is absent from a model, a parameter estimate and error of zero is substituted in the model and the model-averaged regression coefficient for that predictor is estimated by averaging over all best models[54]. We only report the results for the spatial scale of 1000m as results are similar at all investigated scales.

We used data from the nine additional sites sampled in 2016–17 to assess if the best models (or model-averaged results) developed for the 2015–16 data predicted species richness and activity for the 2016–17 data well. We obtained the predicted values of species richness and activity based on standardized and centered values of the predictor variables for these nine additional coffee sites [51], and calculated confidence interval bounds for the predicted species richness and activity by substituting the lower and upper confidence limits for the parameter estimates in the model [55].

### Species-environment associations

Finally, we used canonical correspondence analysis (CCA) to examine how bat species composition at the assemblage-level changed across sites in relation to changes in predictor variables [56]. We also tested for the significance of these associations using the mock ANOVA function in the vegan Package [57]. In addition, we assessed the degree of species turnover in the 20 sites that were sampled in multiple years. We used the software COMDYN [58] to estimate the species turnover parameter GAMMA, defined as the proportion of sample 2 species present in sample 1. COMDYN uses capture-recapture models for closed animal populations that consider heterogeneous detection probabilities of species in the community [58].

All analyses except species richness estimation and turnover were carried out using R 3.4.2 [59].

## Results

Our sites were located at a wide range of distances from natural forests (93–8258 m, mean 1667 m, sd 2246 m). Tree density in sites ranged from 163 trees/ha to 969 trees/ha (mean 482.9, sd 250.9), while the proportion of built up area around sites ranged from 0.006 to 0.1, with the largest value being at a site at very close proximity to a town (mean 0.009, sd 0.018).

Across all the 29 sites, we distinguished nine species/phonic types during the study period (Table 1, S2 Fig). Of the nine, four belonging to the families Rhinolophidae and Hipposideridae (*Rhinolophus lepidus*, *Rhinolophus indorouxii*, *Rhinolophus beddomei*, *Hipposideros sp*., an unknown Rhinolophid) were labelled as cluttered space foragers. The remaining five (P22, P30_40, P50 and P60), which were not identified to species level but most likely belonged to the family Vespertilionidae based on the echolocation call shape, were classified as edge space foragers. We did not detect any cluttered space passive gleaning and open space foragers.

**Table 1:** Frequency of observations of each species across all coffee sites.

Family	Species/Phonic type	Foraging Guild	# Nights detected^a^ (20 sites)		# Nights detected^b^ (9 sites)
			2015–16	2016–17	2016–17
Vespertilionidae	P22	Edge-space	39	37	16
Vespertilionidae	P30_40	Edge-space	53	57	37
Vespertilionidae	P50	Edge-space	35	34	34
Vespertilionidae	P60	Edge-space	16	12	6
Rhinolophidae	*Rhinolophus beddomei* (R.bedd)	Cluttered-space	2	6	1
Rhinolophidae	*Rhinolophus indorouxii* (R.indo)	Cluttered-space	13	19	21
Rhinolophidae	*Rhinolophus lepidus* (R.lep)	Cluttered-space	27	30	25
Hipposideridae	*Hipposideros sp*. (Hipp)	Cluttered-space	1	5	8
Rhinolophidae	*Rhinolophus sp*.	Cluttered-space	0	1	2

For 20 sites, data is from 88.5 hours of active Petterson monitoring in each season. For 9 sites, data is from 56.2 hours of active Petterson monitoring in 2016–17

^a.^ 3 nights per site

^b.^ 5 nights per site

### Bat species richness

Estimates of assemblage-level bat species richness for the 20 sites sampled in 2015–16 ranged between 3 and 7 (mean 4.8, sd 1.12). Moran’s I correlogram of estimated species richness did not indicate spatial autocorrelation (S3 Fig). Our model selection exercise indicated comparable support for three models explaining bat species richness. The amount of variance explained by the models was up to 20% and there was no statistically significant effect of any of the considered local- and landscape-level variables on species richness (Table 2).

**Table 2:** Influence of predictor variables on estimated species richness (SR) and activity.

Response	Model*	Estimate (±SE)	AIC_c_	Weight	Adjusted R^2^
SR	~tden	-0.64 (0.65)	62	0.3	0.15
	~builtup	-0.51 (0.62)	62.5	0.24	0.13
	~tden+builtup		63.1	0.18	0.2
Activity	~tden	-0.11 (0.09)	-10.3	0.38	0.13
	~dist	0.05 (0.07)	-8.6	0.16	0.05
	~tden+dist		-8.6	0.16	0.14

*Predictor variables are: dist, distance to nearest natural forest edge (km); tden, mean tree density (#/m^2)^); builtup, proportion of built-up area in a 1000m buffer surrounding a coffee site. Estimates and their standard errors (SE) are from model averaging. All predictor variables are standardized according to [51].

### Bat activity

**Fig 2:**
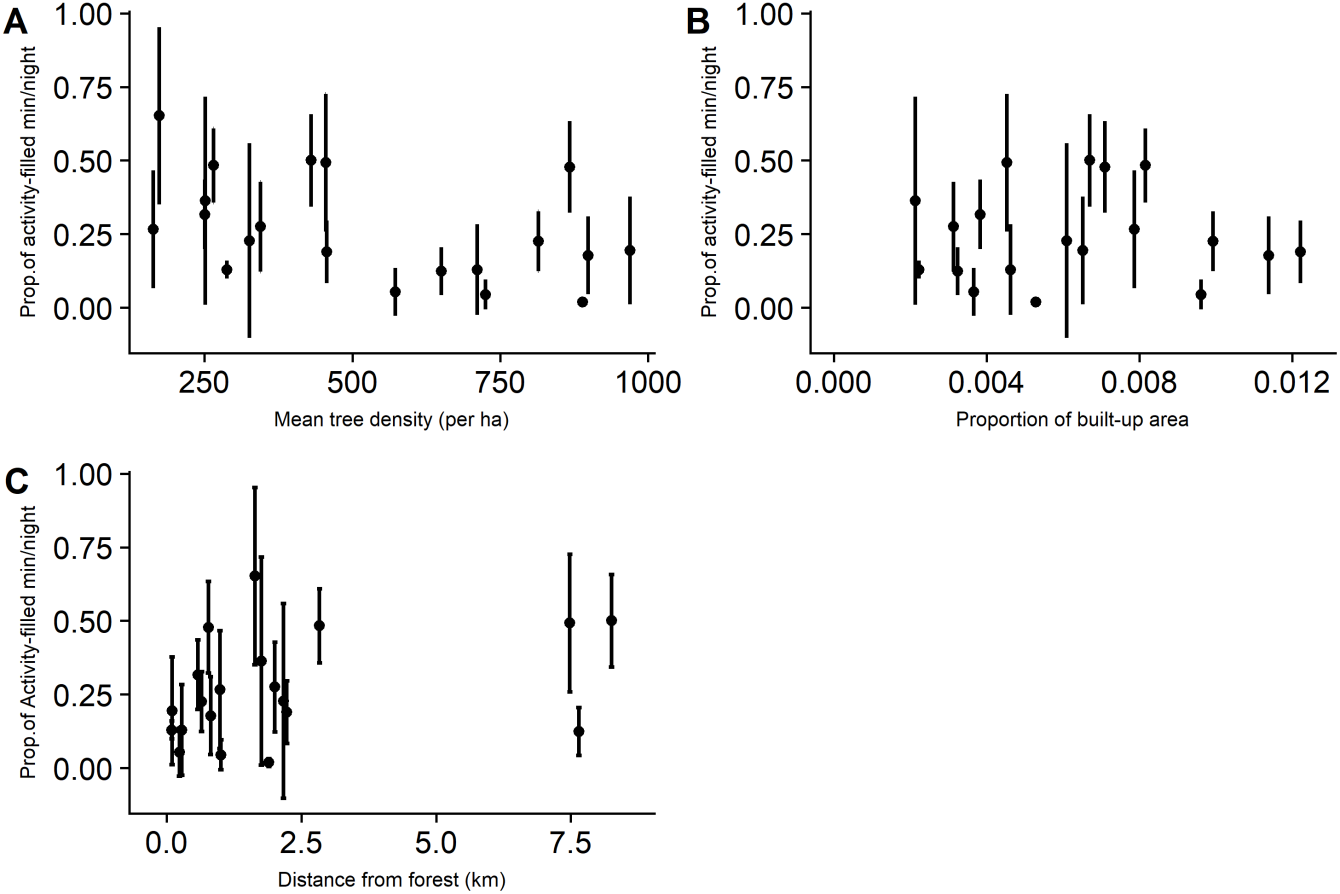
Relationships between mean edge-space forager activity (±SD) and predictor variables. Y axis is proportion.

**Fig 3:**
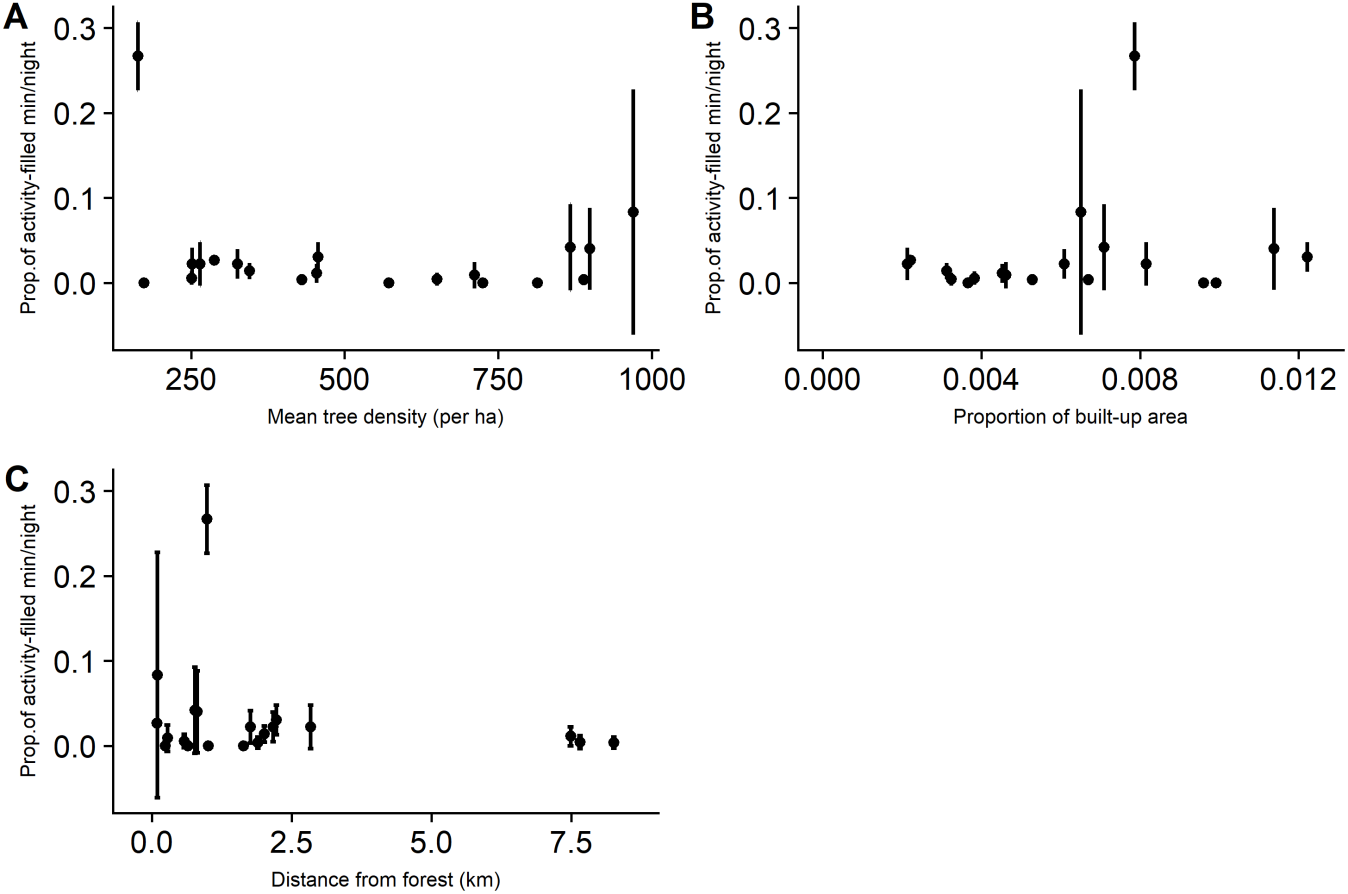
Relationships between mean cluttered-space forager activity (±SD) and predictor variables. Y axis is proportion.

Bat activity levels at the assemblage-level for the 20 sites ranged from 0.02 to 0.65 (mean 0.29, sd 0.18). There was no indication of spatial autocorrelation from the Moran’s I correlogram (S3 Fig). Through model selection, we found comparable support for three models explaining bat activity. The amount of variance explained was up to 15% and none of the predictor variables were statistically significant (Table 2). Ensemble-level activity also showed no apparent relationship with the predictor variables (Figs 2 and 3).

### Species-environment associations

**Fig 4:**
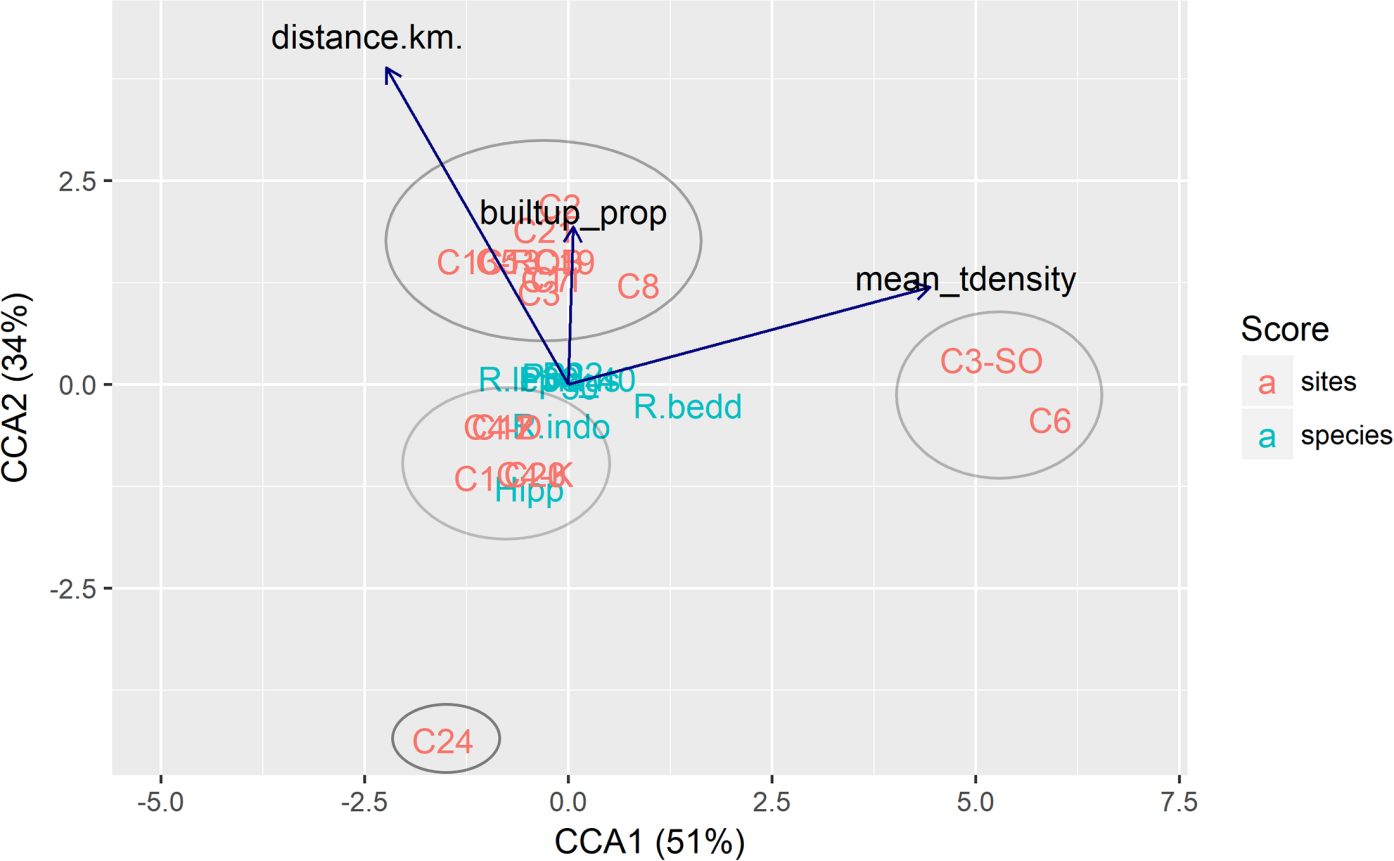
Canonical correspondence analysis (CCA) for insectivorous bat species in 20 coffee sites. Species codes are in Table 1. Length of the arrow denotes the strength of the association between the species and the corresponding predictor variable. Direction of the arrow denotes positive association.

The correspondence analysis identified four clusters of sites that could be visually discriminated (Fig 4). Cluster 1 comprised two sites that were characterized by high tree densities and the presence of *Rhinolopus beddomei* (R.bedd). Clusters 2 (5 sites) and 3 (a single site) occurred closer to natural forest patches, and were characterized by the presence of *Rhinolophus indorouxii* (R.indo) and a *Hipposideros* sp., respectively, which were detected rarely. The remaining sites (cluster 4) were characterized by a higher proportion of built-up area in the neighborhood. Distance of site to the nearest natural forest edge was the most important environmental variable discriminating sites, followed by tree density and proportion of built-up area. However, the correspondence analysis did not reveal any statistically significant relationship between species and the environmental variables considered (p = 0.31; Fig 4).

### Evaluation of modeled relationships

The models developed poorly predicted assemblage-level bat species richness and activity for the additional 9 sites we sampled in 2016–17. The correlation coefficient between predicted and estimated species richness was 0.23, and for bat activity was 0.19. We obtained similar estimates of species richness across years for the 20 sites that were sampled in both years (range 3 to 7 [mean 4.8, sd 1.12] in 2015–16; range 3 to 8 [mean 5.65, sd 1.26] in 2016–17). Species turnover was typically low, with ~85% of the species sampled in a site in 2015–16 also sampled in 2016–17.

## Discussion

### Effect of predictors on species richness

At the site level, we found that tree density had no effect on assemblage-level bat species richness. A potential explanation is that the tree density gradient was not a deterrent for the species detected in this study. Since sun coffee is not grown in the study region, it is difficult to obtain a drastic gradient of tree density, especially at lower end. In a Neotropical study, Bader et al. [60] showed that presence of aerial insectivorous bats was best predicted by canopy cover and canopy height at the local level. Canopy height is largely uniform in the study region. Canopy cover could have led to the observed patterns but we lacked the sample sizes to perform analyses.

At the landscape level, the lack of a clear effect of distance from natural forest areas on species richness is possibly a result of the high tree cover offered by shade coffee in the study area (see Fig 1). Vegetation in the form of trees or hedges can offer increased connectivity in agricultural landscapes for several bat species to exploit resources [61]. In Costa Rica, Mendenhall et al. [22] found that the species richness of bats was similar across forest fragments set in a matrix of coffee farms, concluding that a countryside ecosystem could reduce the contrast between forest areas. No effect of built-up area on species richness likely indicates that the extent of man-made structures in the landscape, while being used by bats, may not be enough to negatively affect species at multiple scales.

### Effect of predictors on bat activity

At the local-level, we found that both assemblage and ensemble-level activity were unaffected by shade tree density. Despite constraints based on wing morphology and echolocation, insectivorous bats are highly flexible [62] and demonstrate variable responses to structural changes in their habitat [63,64]. Edge space foragers may show behavioral flexibility through the use of trails in areas with high clutter levels. For example, Law & Chidel [65] found that a number of bats species sensitive to vegetation clutter use forest tracks and riparian areas as flyways. As expected, we found that clutter-tolerant species did not respond to increase in tree density in the sites. However, we remain cautious about this pattern because the cluttered-space forager activity is dominated by only two of the four species we detected. *Rhinolophus beddomei* (R.bedd) and *Hipposideros sp*. (Hipp) were detected so rarely that it is difficult to ascertain how these species respond to shade management in this region. This supports conclusions of two recent studies from the Western Ghats [17,28] that species with constant frequency echolocation calls like *R*.*beddomei* are at high risk from agricultural intensification.

Insect prey availability at the site level, which was not included as a predictor variable, may also have played a role in the observed patterns [42,66]. Studies show that changes in insect abundance in open areas affect bat activity more strongly than in dense areas [67,68]. This is because prey density is inherently low in open areas and bats adapted to foraging in open spaces respond strongly to any changes in prey density [68]. Since coffee in the study area is grown under a fairly dense canopy (see Methods), insect abundance is likely to have a weak effect on bat activity. Insect abundance can also affect bat activity through its interaction with agricultural practices such as agrochemical use [69]. Organic farming has been shown to increase insect abundance and positively affect bat activity [69]. Alternatively, Poocock & Jennings [70] found bats to be more sensitive to field boundary loss than increased agrochemical inputs. During the time period of our study, coffee is harvested in the region and agrochemical use is minimal. However, pesticides are used through growing season and residual effects of agrochemicals might have impacted the activity of bats.

Increasing distance to forest areas did not affect assemblage and ensemble-level activity of bats. In tree-covered or low-management agricultural landscapes such as this, forest/woodland patches may be less influential for insectivorous bats when compared to homogeneous, intensively managed agriculture [71,72].

### Species-environment associations

Drawing conclusions from species richness alone could mask important trends in compositional changes in bat communities [73]. Through correspondence analysis to elucidate species-environment relationships, we found that most of the species in the study area were not strongly related to the predictor variables and were distributed similarly across the study area. Another explanation for the observed effect of distance from forests could be a limited range of distance values which bats in our study are able to cover. Dispersal distances of bats in Western Ghats are currently unknown [17] but we nevertheless speculate that the low contrast nature of the coffee farms is an important driver of the observed pattern.

### Evaluation of modeled relationships

Our models did not lend to highly accurate predictions of species richness and activity in the new sites. Perhaps this is not very surprising because the models contained non-significant predictors. We also believe that predicting bat activity accurately is a bigger challenge as it may be influenced by other ecological factors such as food availability, presence of roosting sites and high temporal variability of bat activity.

We also found that the complement of species turnover in the sampled sites across the two years was high on an average but we speculate that the low precision was due to the species-poor nature of the assemblage. Wordley [74] also found no annual variation in bat communities in a similar landscape consisting of coffee, tea, forest fragments.

### Conclusions and future research

Our study shows no response of species richness and activity of a Paleotropical bats at both assemblage and ensemble-level to current local shade management practices (characterized by shade tree density and foliage cover) and landscape-level factors in a coffee agroforestry system. However, impacts on particular species might be missed in a low contrast landscape when community level metrics are used as response variables [75]. This is hinted at by our finding that species such as *Rhinolophus beddomei* and *Hipposideros sp*. appear to be rare in our study region. Further research should investigate species-environment relationships in this landscape; especially for such rare species, which may also be vulnerable. Complementary methods like mist netting must also be employed in the future to gather morphological information which has been shown to predict the vulnerability of insectivorous bats to anthropogenic changes [60].

Given that local features on a farm are more easily manipulated than landscape level factors by farmers, we must caution that these neutral responses should not be a reason for large-scale reduction of tree density, as a heterogenous vegetation structure benefits several bat species, particularly ones that have small home ranges [72]. However, farmer decisions in agroforestry systems are subject to non-biological considerations such as commodity price fluctuations, input and labor costs [2] which may be detrimental to biodiversity conservation. To this end, future investigations should quantify levels of biological pest control services across gradients of shade tree densities and other local-level features through manipulative experiments, so that farmers can balance these ecosystems services against economic factors in making decisions that could impact biological diversity.

## Supporting information

S1 Fig Schematic of the Variable Area Transect (VAT) method. The line in the centre is the baseline transect, the rectangles on either side are the cells, open circles are trees. The width of the cells (d1-d8) is the distance of the fifth farthest tree in that cell. The length of each cell is 10m.
(TIF)

S2 Fig Representative call pulses of bats detected in the study. A time expansion detector was used for acoustic monitoring. Therefore, for actual values of time and frequency, divide values of time on x-axis by a factor of 10 and multiply values of frequency on y-axis by a factor of 10.
(TIFF)

S3 Fig Spatial correlograms of estimated species richness and activity. Error bars denote standard deviations.
(TIFF)

S1 Dataset Acoustic recording data and predictor variables in Chikmagalur district. Observed and estimated species richness, total and guildwise bat activity, predictor variables at each of the 20 sites sampled in 2015–16. Predicted and estimated species richness, predicted and observed total activity, predictor variables at each of the 9 sites sampled in 2016–17.
(XLSX)
