# Supplementary figures and images for: Responses of aerial insectivorous bats to local and landscape-level features of coffee agroforestry systems in Western Ghats, India
Source: PLoS One. 2018 Aug 16;13(8):e0201648. doi: 10.1371/journal.pone.0201648 (PMC6095497; doi:10.1371/journal.pone.0201648)

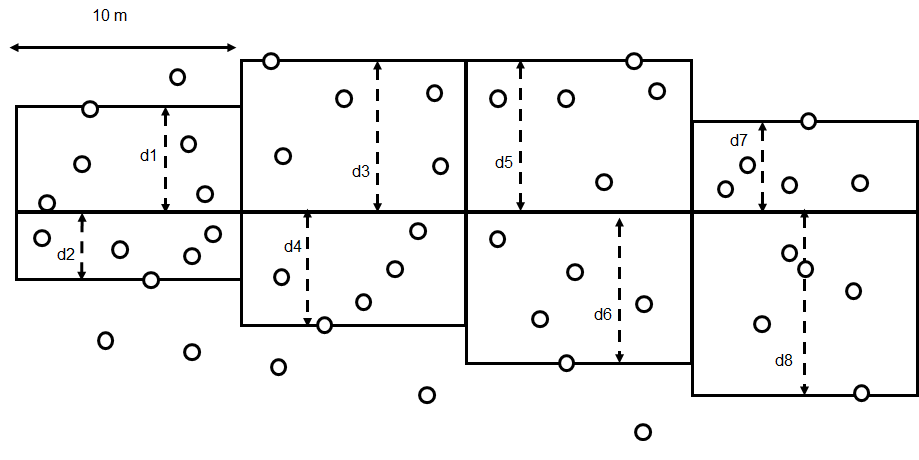

Supplement: S1 Fig — The line in the centre is the baseline transect, the rectangles on either side are the cells, open circles are trees. The width of the cells (d1-d8) is the distance of the fifth farthest tree in that cell. The length of each cell is 10m. (TIF) [file pone.0201648.s001.tif]

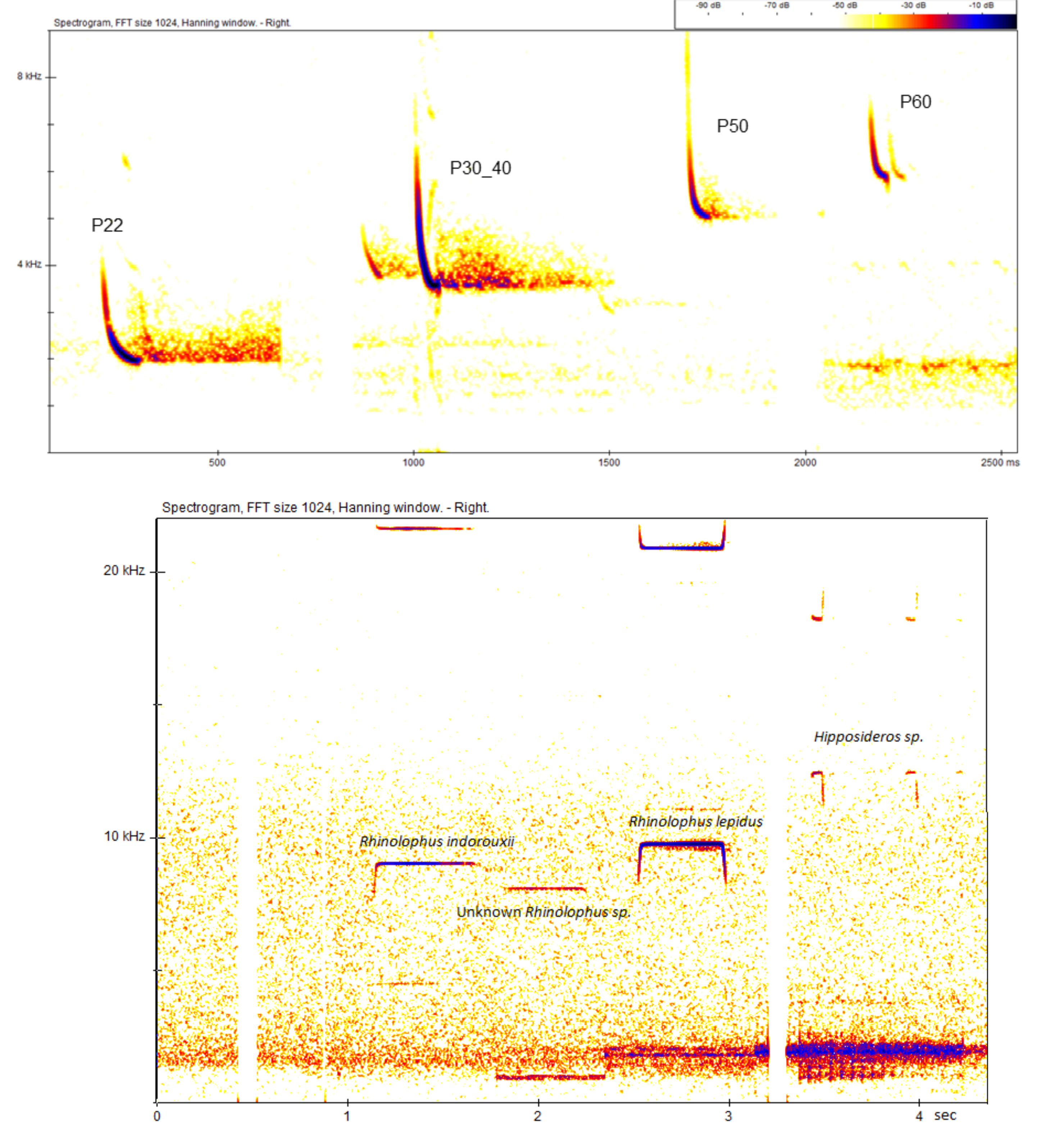

Supplement: S2 Fig — A time expansion detector was used for acoustic monitoring. Therefore, for actual values of time and frequency, divide values of time on x-axis by a factor of 10 and multiply values of frequency on y-axis by a factor of 10. (TIFF) [file pone.0201648.s002.tiff]

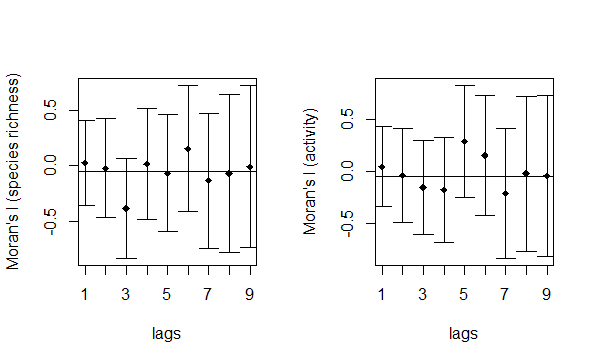

Supplement: S3 Fig — Error bars denote standard deviations. (TIFF) [file pone.0201648.s003.tiff]
